# A modular and optimized single marker system for generating *Trypanosoma brucei* cell lines expressing T7 RNA polymerase and the tetracycline repressor

**DOI:** 10.1098/rsob.110037

**Published:** 2012-02

**Authors:** S. K. Poon, L. Peacock, W. Gibson, K. Gull, S. Kelly

**Affiliations:** 1 Sir William Dunn School of Pathology, University of Oxford, South Parks Road, Oxford OX1 3RE, UK; 2 Oxford Centre for Integrative Systems Biology, Department of Biochemistry, University of Oxford, South Parks Road, Oxford OX1 3QU, UK; 3 School of Biological Sciences, University of Bristol, Bristol BS8 1UG, UK; 4 Centre for Mathematical Biology, Mathematical Institute, University of Oxford, 24-29 St Giles’, Oxford OX1 3LB, UK; 5 Department of Plant Sciences, University of Oxford, South Parks Road, Oxford OX1 3RB, UK

**Keywords:** trypanosomatid, expression, optimization, inducible, codon, tsetse

## Abstract

Here, we present a simple modular extendable vector system for introducing the T7 RNA polymerase and tetracycline repressor genes into *Trypanosoma brucei*. This novel system exploits developments in our understanding of gene expression and genome organization to produce a streamlined plasmid optimized for high levels of expression of the introduced transgenes. We demonstrate the utility of this novel system in bloodstream and procyclic forms of *Trypanosoma brucei*, including the genome strain TREU927/4. We validate these cell lines using a variety of inducible experiments that recapture previously published lethal and non-lethal phenotypes. We further demonstrate the utility of the single marker (SmOx) TREU927/4 cell line for *in vivo* experiments in the tsetse fly and provide a set of plasmids that enable both whole-fly and salivary gland-specific inducible expression of transgenes.

## 2. Introduction

The trypanosomatids are a group of unicellular eukaryotes, several of which cause globally important parasitic diseases of humans and livestock. In sub-Saharan Africa, one species of trypanosomatid, *Trypanosoma brucei*, causes human African trypanosomiasis and the cattle disease nagana, which together impose a huge burden on human health and welfare. In addition to its importance as the etiological agent of a neglected disease of the developing world, *T. brucei* is also a model organism for studying a large variety of biological processes, including antigenic variation [1], the eukaryotic cilium and flagellum [2], glycosylphosphatidylinositol anchors [3] and RNA editing [4]. As such, the trypanosomatids are the focus of much attention from disparate academic communities and have provided a wealth of insight into many fundamental aspects of biology.

The ability to perform experiments on trypanosomatids was revolutionized by the introduction of cell lines that stably expressed both the T7 RNA polymerase and the tetracycline repressor protein (29 : 13, S16 and 13 : 90 cell lines) [5]. These cell lines have provided a platform for the trypanosome research community for over 10 years, facilitating advancement of our understanding of the disease and many aspects fundamental to eukaryotic biology. Additional cell lines expressing the T7 RNA polymerase, the tetracycline repressor or both have also been generated [6–8]. These have opened up further avenues of interrogation and experimentation within the tsetse fly [8].

There is a need to be able to easily extend the range of trypanosomes in which one can do inducible experimentation. For example, there are currently no inducible cell lines for performing experiments in the human-infective *Trypanosoma brucei gambiense* or *Trypanosoma brucei rhodesiense*. Moreover, there is also no inducible version of the genome strain TREU927/4 [9].

The ability to use novel cell lines and perform inducible experimentation in disparate strains and novel field isolates will be critical to future trypanosomatid research in a post-genomic era. To pre-empt this emerging need, we analysed and exploited the available genome information to develop a single marker modular vector system for introducing the T7 polymerase and tetracycline repressor genes into any trypanosomatid species. We demonstrate that this system works for the genome strain TREU927/4 and Lister 427 (bloodstream and procyclic) *T. brucei* parasites. We also show that TREU927/4-derived cells can be used equally well for *in vitro*- and *in vivo*-inducible experiments in tsetse flies.

## 3. Material and methods

### 3.1. Plasmid construction

The Single Marker Oxford (SmOx) plasmid was constructed *de novo* using a combination of the Paraflagellar rod protein 2 (PFR2) intergenic sequences amplified from TREU927/4 genomic DNA, synthetic codon-optimized genes (MrGene, Invitrogen) and the necessary bacterial origin of replication and ampicillin-resistance gene from pDex-577 [7]. PFR2 intergenic sequences were selected as they are short and contain well-characterized trans-splicing and polyadenylation sites [10]. Codon optimization was performed as it has been previously reported that translational selection may impact on protein expression in trypanosomatids [11]. Codon selection for the transgenes was made based on a bioinformatic analysis of all available trypanosomatid genomes, so that the transgenes would use codons that are frequently used in all trypanosomatids. The plasmid was sequenced to 3X coverage using the EZ-Tn5^TM^ KAN-2 Insertion kit (Epicentre Technologies Co.). The vector sequence is provided as electronic supplementary material, file S1. For transfection into *T. brucei* cells, the vector was linearized by restriction digest with *Hind*III, which excises the bacterial component of the plasmid so that only the desired transgenes and necessary intergenic regions are integrated into the genome. For transfection, 10 μg of the SmOx plasmid was linearized with *Hind*III and transfected into each of TREU927/4, Lister 427 procyclic and Lister 427 bloodstream forms to create SmOxP927, SmOxP427 and SmOxB427, respectively. To calculate the mean doubling time for each cell line, cells were sub-cultured every 24 h for 5 days to the same cell density (1 × 10^6^ or 1 × 10^5^ cells ml^−1^ for procyclic and bloodstream form, respectively). The mean and standard deviation were computed treating each 24 h period as replicates.

### 3.2. Codon optimization

All genome data were retrieved from TriTrypDB.org [12]. Codon usage statistics were computed using a method previously described [13]. In brief, the Carbone codon adaptation index (cCAI) score [13] is an optimized codon adaptation index score that uses the genes that display the strongest codon bias to compute a codon usage matrix and then re-scores every other gene according to this matrix; iterations of this process lead to identification of the most commonly used codons in any genome [13]. Given that codon usage is intrinsically liked to gene mRNA levels in all organisms, we chose to optimize the T7 RNA polymerase and tetracycline repressor for expression in trypanosomatids using a consensus of optimal codons computed from the available trypanosomatid genomes. Genes were re-encoded to use the codons that occur most frequently in trypanosomatids and synthesized (MrGene, Invitrogen).

### 3.3. Inducible transgene expression

For each cell line, 10 μg pDex777-GFP plasmid (the sequence is provided as electronic supplementary material, file S2) was linearized with *Not*I restriction endonuclease and transfected into SmOxP427, SmOxP927, 29 : 13 [5], SmOx2B427 and S16 [5] cells. Cells were subsequently selected with 1 µg ml^−1^ puromycin and 5 µg ml^−1^ phleomycin (Sigma-Aldrich). After selection, cell lines were removed from puromycin and phleomycin for 48 h with sub-culturing of cells to either 1 × 10^6^ or 1 × 10^5^ every 24 h for procyclic and bloodstream-form cells, respectively. At 48 h, GFP expression was induced by the addition of 1 µg ml^−1^ doxycycline (Sigma-Aldrich) to the culture medium. For flow cytometry analysis, four independent clones of each cell line were selected at random following the transfection of the pDex-777 plasmid. As above cell lines were sub-cultured every 24 h to the same cell density (1 × 10^6^ or 1 × 10^5^ cells ml^−1^) for 48 h before induction. Green fluorescent protein (GFP) expression was induced by the addition of 1 µg ml^−1^ doxycycline to culture medium. Twenty-four hours post-induction, cells were fixed by the addition of paraformaldehyde directly to the culture medium and incubating for 10 min. The final concentration of paraformaldehyde was 1 per cent. Cells were then washed and resuspended in PBS. Data were acquired with a FACSCalibur flow cytometer (BD Biosciences).

### 3.4. Inducible transgene expression in tsetse flies

For assaying inducible transgene expression in the tsetse fly, procyclic form trypanosomes of a single clone of each of TREU927/4, SmOxP927, SmOxP927 containing the pDex-777 plasmid and SmOxP927 cells containing the pDex-577 [7] plasmid were fed to individual groups of tsetse (*Glossina* sp.) via a silicone membrane, as previously described [14]. Flies were maintained at 25°C, 70 per cent relative humidity and fed on sterile horse blood supplemented with 2.5 per cent wt/vol BSA [15] and 1 mM dATP [16]. Thirty days post-infection flies were starved for 2 days and then fed on horse serum containing tetracycline (25 μg ml^−1^) for 20 min. Two days later, the midgut, proventriculus and salivary glands were dissected separately into PBS and inspected by fluorescence microscopy for the presence of GFP fluorescent trypanosome cells.

### 3.5. Inducible RNA interference

Inducible RNA interference in the SmOx cell lines was tested using the PFR2 RNAi vector [17]. For each cell line, 10 μg of PFR2 RNAi plasmid DNA was linearized with *Not*I restriction endonuclease and transfected into SmOxP427, SmOxP927 and SmOxB427 cells. Cells were then selected with 1 µg ml^−1^ puromycin and 5 µg ml^−1^ phleomycin. Following selection, cell lines were removed from any puromycin and phleomycin for 48 h. Cells were then sub-cultured every 24 h to the same cell density (1 × 10^6^ or 1 × 10^5^ cells ml^−1^ for procyclic and bloodstream form, respectively). After 48 h, RNAi was induced by the addition of 1 µg ml^−1^ doxycycline to the culture medium. Cell density and cell size measurements were performed using CASY model TT cell-counter (Innovatis). For western blot analysis, protein samples were collected every 24 h during the 96 h induction. Transmission electron microscopy analysis was performed on cells that had been induced for 72 h. At 96 h post-induction, cells were spun down, washed in fresh medium and recovered in medium without doxycycline for 24 h to determine if cells could be de-induced. For western blot analysis of protein levels 5 × 10^6^ cells from each time point for each cell line were loaded onto a 10 per cent sodium dodecyl sulphate polyacrylamide gel electrophoresis (SDS-PAGE) gel. Following transfer, PFR2 protein was detected using the anti-PFR2 antibody L8C4 [18] at a 1 : 1000 dilution. Rabbit anti-mouse IgG (whole molecule)-peroxidise-conjugated antibody (Sigma-Aldrich) at 1 : 20 000 dilution was used for ECL detection using standard protocols.

## 4. Results

### 4.1. A modular single marker vector (SmOx) for generation of trypanosome cell lines expressing T7 RNA polymerase and the tetracycline repressor protein

**Figure 1.**
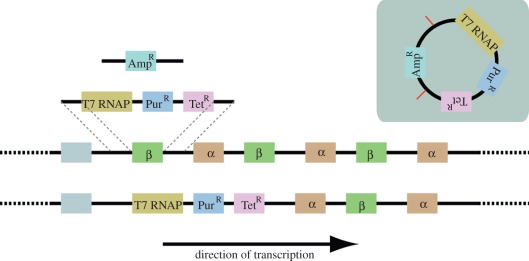
Cartoon of the genome before and after integration of the SmOx plasmid. β is the β-tubulin gene. α is the α-tubulin gene. T7 RNAP is the T7 RNA polymerase. Pur^R^ is the puromycin-resistance gene. Tet^R^ is the tetracycline repressor gene. Amp^R^ is the ampicillin-resistance gene encoded on the bacterial part of the plasmid. Grey inset contains cartoon of plasmid prior to restriction digest with *Hind*III. Restriction sites indicated by red bars.

A simple modular plasmid was designed to integrate into the tubulin gene locus replacing the first β-tubulin gene. This locus has been repeatedly used by trypanosome researchers as a reliable genomic location to generate read-through transcription and is the same location used in previous inducible strains [5]. We designed the vector so that, upon linearization, the bacterial component of the plasmid is excised and only the desired coding sequences and intergenic sequences are integrated into the genome (figure 1). The plasmid is designed to exploit the fact that trypanosomes employ polycistronic transcription for transcription of protein-coding genes, and hence there are no promoter sequences included in the plasmid. The plasmid was also designed to integrate cleanly into the trypanosome genome. The strategy employed was to replace the first β-tubulin gene (from start codon to stop codon) in the tubulin gene array on chromosome 1. No gene truncations or partial gene fragments were created in this integration (figure 1). A previously validated nuclear localization sequence from the trypanosome LA protein [19] was added to the N-terminus of both the T7 RNA polymerase and the Tet repressor gene to facilitate nuclear targeting of the expressed transgenes. To enhance translational efficiency and enable the broadest possible use for the transgenes in trypanosomatids, codon optimization of the transgenes was performed based on an analysis of codon usage frequency in all trypanosomatid genomes (table 1).

**Table 1:** Table of codon usage in available trypanosomatid genomes. For each amino acid, codons are presented in descending order according to their mean codon usage score across all trypanosomatid genomes. The most commonly occurring codon for each amino acid is given a score of 1. The score for each subsequent codon represents the frequency of occurrence relative to the most common codon (e.g. a score of 0.5 indicates that this codon is used 50% less frequently than the most frequently used codon for this amino acid).

amino acid	codon	*Trypanosoma vivax*	*Trypanosoma brucei gambiense*	*Trypanosoma brucei*	*Trypanosoma congolense*	*Trypanosoma cruzi*	*Leishmania mexicana*	*Leishmania major*	*Leishmania infantum*	*Leishmania braziliensis*
A	GCG	1.00	0.91	0.81	0.99	1.00	1.00	1.00	1.00	1.00
	GCC	0.76	0.79	0.72	0.91	0.76	0.83	0.79	0.83	0.84
	GCA	1.00	1.00	1.00	1.00	0.77	0.47	0.45	0.46	0.59
	GCT	0.71	0.93	0.78	0.90	0.60	0.42	0.40	0.41	0.51
C	TGC	1.00	0.96	1.00	1.00	1.00	1.00	1.00	1.00	1.00
	TGT	0.75	1.00	0.90	0.90	0.79	0.30	0.29	0.27	0.37
D	GAC	1.00	0.79	0.86	0.94	0.91	1.00	1.00	1.00	1.00
	GAT	0.82	1.00	1.00	1.00	1.00	0.43	0.42	0.43	0.48
E	GAG	1.00	1.00	1.00	1.00	1.00	1.00	1.00	1.00	1.00
	GAA	0.67	0.83	0.91	0.70	0.69	0.24	0.24	0.24	0.28
F	TTC	0.69	0.72	0.75	0.73	0.42	1.00	1.00	1.00	1.00
	TTT	1.00	1.00	1.00	1.00	1.00	0.57	0.55	0.55	0.66
G	GGC	1.00	0.64	0.73	0.91	1.00	1.00	1.00	1.00	1.00
	GGT	0.85	1.00	1.00	1.00	0.84	0.39	0.37	0.35	0.47
	GGG	0.70	0.65	0.65	0.81	0.77	0.39	0.36	0.34	0.41
	GGA	0.62	0.68	0.76	0.77	0.74	0.21	0.19	0.19	0.26
H	CAC	1.00	1.00	1.00	1.00	1.00	1.00	1.00	1.00	1.00
	CAT	0.66	0.86	0.84	0.71	0.90	0.33	0.32	0.33	0.36
I	ATC	0.65	0.60	0.61	0.66	0.54	1.00	1.00	1.00	1.00
	ATT	1.00	1.00	1.00	1.00	1.00	0.46	0.44	0.45	0.57
	ATA	0.49	0.54	0.60	0.53	0.33	0.15	0.15	0.15	0.19
K	AAG	1.00	1.00	1.00	1.00	1.00	1.00	1.00	1.00	1.00
	AAA	0.63	0.78	0.94	0.74	0.74	0.21	0.20	0.21	0.25
L	CTG	1.00	0.80	0.86	1.00	100	1.00	1.00	1.00	1.00
	CTC	0.72	0.69	0.71	0.78	0.54	0.68	0.65	0.67	0.70
	CTT	0.90	1.00	1.00	0.97	0.88	0.30	0.29	0.30	0.36
	TTG	0.78	0.88	0.90	0.87	0.88	0.30	0.29	0.29	0.33
	CTA	0.33	0.35	0.45	0.34	0.20	0.13	0.12	0.13	0.17
	TTA	0.28	0.45	0.49	0.41	0.30	0.05	0.04	0.04	0.07
M	ATG	1.00	1.00	1.00	1.00	1.00	1.00	1.00	1.00	1.00
N	AAC	1.00	1.00	1.00	1.00	0.95	1.00	1.00	1.00	1.00
	AAT	0.75	0.94	0.89	0.90	1.00	0.27	0.26	0.26	0.35
P	CCG	0.89	0.84	0.80	0.97	1.00	1.00	1.00	1.00	1.00
	CCA	1.00	1.00	1.00	1.00	0.91	0.44	0.40	0.41	0.58
	CCC	0.72	0.84	0.77	0.90	0.69	0.50	0.48	0.48	0.57
	CCT	0.74	0.83	0.77	0.82	0.67	0.36	0.33	0.35	0.45
Q	CAG	1.00	1.00	1.00	1.00	1.00	1.00	1.00	1.00	1.00
	CAA	0.64	0.82	0.88	0.67	0.66	0.24	0.23	0.23	0.28
R	CGC	1.00	0.90	0.93	1.00	0.92	1.00	1.00	1.00	1.00
	CGT	0.76	1.00	1.00	0.90	1.00	0.34	0.33	0.32	0.40
	CGG	0.56	0.77	0.79	0.79	0.79	0.45	0.43	0.43	0.44
	AGG	0.60	0.64	0.69	0.75	0.63	0.19	0.18	0.17	0.22
	CGA	0.47	0.57	0.61	0.56	0.61	0.25	0.23	0.23	0.29
	AGA	0.41	0.43	0.52	0.50	0.41	0.09	0.09	0.09	0.12
S	AGC	1.00	0.88	0.98	1.00	1.00	1.00	1.00	1.00	1.00
	TCG	0.72	0.80	0.77	0.78	0.91	0.83	0.84	0.83	0.80
	TCC	0.66	0.86	0.81	0.85	0.98	0.68	0.65	0.64	0.67
	TCT	0.69	0.90	0.84	0.76	0.94	0.41	0.39	0.40	0.47
	AGT	0.77	1.00	1.00	0.88	0.88	0.31	0.28	0.29	0.39
	TCA	0.67	0.93	0.93	0.82	0.85	0.32	0.29	0.29	0.40
T	ACG	0.92	0.86	0.76	0.95	1.00	1.00	1.00	1.00	1.00
	ACA	1.00	1.00	1.00	1.00	0.78	0.43	0.42	0.41	0.57
	ACC	0.62	0.72	0.65	0.80	0.57	0.74	0.72	0.70	0.82
	ACT	0.63	0.78	0.66	0.73	0.50	0.29	0.28	0.28	0.42
V	GTG	1.00	1.00	1.00	1.00	1.00	1.00	1.00	1.00	1.00
	GTT	0.65	0.82	0.82	0.69	0.52	0.24	0.23	0.23	0.27
	GTC	0.40	0.41	0.42	0.41	0.35	0.52	0.50	0.54	0.51
	GTA	0.32	0.44	0.48	0.36	0.23	0.15	0.14	0.15	0.20
W	TGG	1.00	1.00	1.00	1.00	1.00	1.00	1.00	1.00	1.00
Y	TAC	1.00	1.00	1.00	1.00	1.00	1.00	1.00	1.00	1.00
	TAT	0.66	0.83	0.82	0.72	0.62	0.21	0.20	0.20	0.25
STOP	TGA	1.00	1.00	0.96	1.00	1.00	1.00	1.00	1.00	1.00
	TAG	0.73	0.73	0.66	0.69	0.24	0.80	0.85	0.82	0.84
	TAA	0.64	0.94	1.00	0.91	0.37	0.46	0.46	0.45	0.54

### 4.2. T7 polymerase and Tet repressor transgenes are expressed

**Figure 2.**
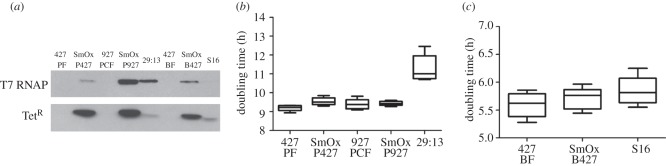
Expression and growth analysis of SmOx cell lines. (*a*) Western blots showing the expression of T7 RNA polymerase and tetracycline repressor proteins in all SmOx cell lines. For comparison, 29 : 13 and S16 cells are also shown. (*b*) Growth rate of SmOx procyclic-form cell lines compared with their parental cell lines. (*c*) Growth rate of the SmOx bloodstream form cell line compared with its parental cell line. In all cases, PF is procyclic form and BF is bloodstream form. Error bars indicate the spread of the data. Loading controls for western blots are provided as the electronic supplementary material, file S3.

To determine whether the introduced transgenes resulted in the production of expressed protein, we analysed the expression of the T7 polymerase and Tet repressor by western blot (figure 2*a*). This analysis showed that both the transgenes were expressed. Moreover, the Tet repressor gene was expressed to higher levels than in previous cell lines, presumably through the combined effects of codon optimization and the use of different UTR sequences (figure 2*a*). T7 RNA polymerase expression appears lower in SmOxP427 cells than in SmOxP927 or 29 : 13. In contrast to this, T7 RNA polymerase expression in SmOxB427 was substantially higher than in S16. While some variation across strains was observed, no variation within strains could be detected by western blot analysis of multiple independent clones (data not shown). The average doubling time of the novel SmOx cell lines did not differ from their parental cell lines (figure 2*b*,*c*). Also, there was no detectable difference in cell volume or cell morphology (data not shown).

### 4.3. Cell lines function in inducible transgene tests

**Figure 3.**
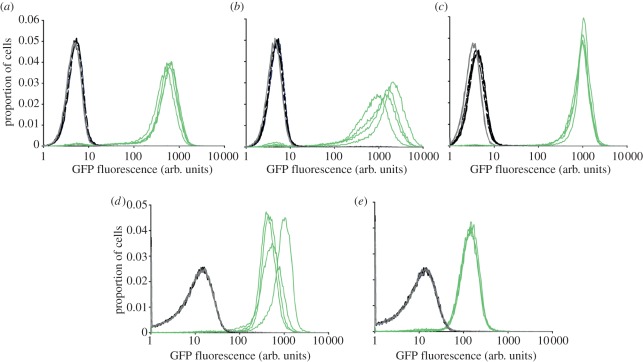
Comparison of inducible transgene expression between cell lines. In each case, four independent clones of pDex-777-containing cell lines were induced for 24 h. Flow cytometry measurements using identical settings were performed on induced (green lines) and non-induced (black lines) parental non-transfected clones (grey lines). (*a*) SmOxP427. (*b*) SmOxP927. (*c*) 29 : 13. (*d*) SmOxB427. (*e*) S16.

Having determined that the SmOx cell lines expressed the desired transgenes and demonstrated no observable growth defects, a subsequent test was designed to determine whether the newly created cell lines were capable of tetracycline-inducible regulation of gene expression. The pDex-777 plasmid (electronic supplementary material, file S2) was used. This plasmid integrates into the mini chromosomes and encodes a GFP gene under the control of a T7 RNA polymerase promoter. Three tetracycline operators are positioned between the T7 promoter and the GFP-coding sequence, allowing tetracycline-dependent control of transgene expression. Four independent clones for each of the three SmOx cell lines were induced for 24 h, and GFP expression was analysed by flow cytometry (figure 3). This revealed that for procyclic-form SmOxP427 (figure 3*a*) and SmOxP927-(figure 3*b*), inducible GFP expression was comparable with that observed in 29 : 13 (figure 3*c*). For both SmOxP427 and SmOxP927, non-induced cells were less fluorescent compared with parental no vector control than the equivalent 29 : 13 cells. This suggests that the non-induced state in the SmOx cells is more transcriptionally silent than in 29 : 13. However, SmOxP427 (figure 3*a*) and SmOxP927 (figure 3*b*) demonstrated more variability between clones in expression level obtained, with the mean expression in SmOx927 being higher than in 29 : 13 and the mean expression in SmOxP427 being lower than in 29 : 13 cells. Similarly, SmOxB427 cells exhibited more variability between clones, though expression in all clones was higher than that observed for S16 cells (figure 3*d*,*e*). No difference could be detected between the parental and non-induced bloodstream or procyclic-form SmOx cell lines, demonstrating that the non-induced cells have no detectable expression of GFP.

### 4.4. Cell lines function in RNA interference tests

**Figure 4.**
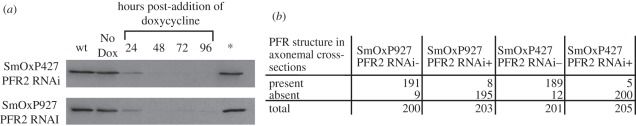
Analysis of RNAi phenotypes by a variety of methods. (*a*) Western blot analysis of the Paraflagellar rod protein 2 (PFR2) protein levels following induction of PFR2 RNAi in SmOx P427 and SmOx P927 cell lines. wt is the parental strain not containing the integrated SmOx plasmid. No Dox is the non-induced control. Asterisk (*) indicates cells that, following 96 h of induction of RNAi, were pelleted, washed and resuspended in fresh medium in the absence of doxycycline and allowed to recover for 24 h. (*b*) Electron microscopy analysis of cross sections of axonemes. Individual axonemes were scored for the presence or the absence of a recognizable PFR structure. Loading controls for western blots are provided as electronic supplementary material, file S3.

To determine whether RNA interference using established RNAi tools [20] was also possible, we evaluated the ability of the SmOxP427, SmOxP927 and SmOxB427 to recapitulate a previously characterized RNAi phenotype [17]. We selected the PFR2 gene [17] for this purpose as there is a defined and readily observable phenotype in procyclic-form cells and it is lethal in bloodstream-form cells. The same PFR2 RNAi plasmid was used as described previously [17] and transfected into each of SmOxP427, SmOxP927 and SmOxB427. In all cases, the observed phenotypes were as expected. In both the procyclic-form SmOx cell lines, there was a reduction in PFR2 protein levels observed by western blot (figure 4*a*). At 72 h post-induction, SmOxP427 and SmOxP927 cells were analysed by transmission electron microscopy for the presence of the PFR structure. The number of axonemal cross-sections which had no observable PFR structure associated with the axoneme was recorded (figure 4*b*). In agreement with previous experiments using 29 : 13 cells, there was a dramatic loss of the PFR structure upon induction of RNAi against PFR2, and induction of PFR2 RNAi in the bloodstream form was lethal within 24 h (data not shown).

### 4.5. Genome strain-derived SmOxP927 cells can be used for *in vivo* studies in tsetse flies

**Figure 5.**
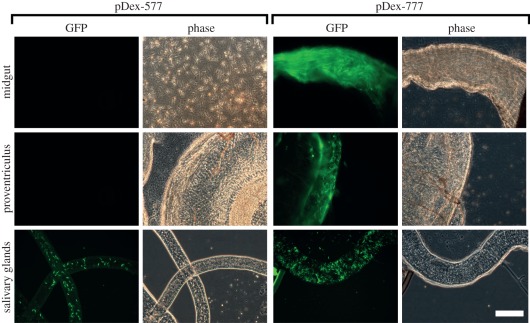
Inducible transgene expression in SmOxP927 cells in different compartments of the tsetse fly. Forty-eight hours-induced cells are shown for both the pDex-577 and pDex-777 plasmid. All images taken at the same magnification; scale bar indicates 50 μm. Representative GFP fluorescence (GFP) and phase contrast (Phase) images are shown for each infected compartment of the fly. The midgut image for pDex-577 shows trypanosome cells that have spilled out of the dissected midgut. The midgut image for pDex-777 shows a midgut full of fluorescent trypanosome cells, in which it is difficult to resolve individual cells. The proventriculus images for both pDex-577 and pDex-777 show an infected proventriculus with trypanosome cells. The salivary gland images for pDex-577 and pDex-777 show an infected salivary gland.

Having demonstrated the functionality of SmOx cell lines under cell culture conditions, we evaluated the newly generated SmOxP927 cell line for inducible experimentation in tsetse flies. SmOxP927 cells were transfected with one or other of two tetracycline-inducible plasmids pDex-577 [7] and pDex-777. These two plasmids are identical in sequence except for the 3′UTR immediately downstream of the inducible transgene. pDex-577 has an aldolase 3′UTR and pDex-777 has PFR2 3′UTR immediately downstream of the GFP-coding sequences. Procyclic forms of each cloned SmOxP927 cell line were used to infect tsetse flies. Thirty days post-feeding, GFP was induced by feeding half of the infected tsetse flies with horse serum containing tetracycline and the other half was fed horse serum without tetracycline as a non-induced control. Two days later 66 flies were dissected, of which 48 had a midgut infection, 46 had an infected preventriculus and 24 had a salivary gland infection. There were no observed differences in infection rate between induced and non-induced cells. No GFP-positive *T. brucei* cells were found in either parental or non-induced infected flies. Interestingly, upon induction, the two plasmids used produced dramatically different expression patterns within the fly (figure 5). For pDex-577, GFP fluorescent trypanosomes were observed only in the salivary glands. Trypanosome cells infecting the midgut and proventriculus had no detectable GFP fluorescence (figure 5). In contrast, induction of pDex-777 produced green fluorescent trypanosome cells in all three compartments of infected flies (figure 5), including proventricular forms.

## 5. Discussion

We present a novel single marker plasmid that integrates into a defined locus to produce *T. brucei* cells expressing the T7 RNA polymerase and the tetracycline repressor gene. This work presents advancement over existing technologies in the light of recent genomic data. The design of the plasmid is streamlined and modular, allowing future elaboration and modification. We demonstrate the utility of this plasmid in two independent strains and life-cycle stages of *T. brucei*. In all tests, the cell lines created perform as well as or better than pre-existing laboratory strains. Under normal cell culture conditions, these cell lines grow without any apparent growth defect, they have normal morphology, they express high levels of the desired transgenes in both procyclic- and bloodstream-form cells, and they exhibit both high inducibility and tight repression of expression. In side-by-side tests under normal growth conditions, they also grow faster than pre-existing inducible cell lines, allowing for more rapid selection of transformed cells. Furthermore, requirement for only a single selectable marker renders more selectable markers available for use.

We show that these cell lines are capable of both inducible transgene expression and inducible RNAi, and recapitulate both lethal and non-lethal phenotypes previously published using other strains. We also provide further validation of the genome strain-derived SmOxP927 cell line by demonstrating that this cell line can be used for tetracycline-inducible experimentation in the tsetse fly as well as in routine cell culture. This novel feature will enable more rapid transition from *in vitro* to *in vivo* experimentation in the future. By using two different plasmids, which differ only in the 3′UTR associated with the reporter gene, we also provide an additional inducible expression resource demonstrating that it is possible to perform compartment-specific-inducible gene expression within the tsetse fly. This result indicates that careful selection of 3′UTR sequences may be important for *in vivo* experiments in tsetse flies.

In addition to the benefits of using a single-resistance marker and having both high inducibility and low leakiness, this single marker vector system will also increase the capability of the trypanosomatid research community by facilitating the use of novel strains and field isolates of trypanosomatids for inducible experimentation. This system will also enable continued used of pre-existing expression technologies dependent on both T7 RNA polymerase and the tetracycline repressor, and will therefore further the spread of these powerful technologies within the field. Future developments of this modular system could exploit additional repressor proteins or use the Cre-Lox recombinase that has been previously used successfully in trypanosomatids [21] to enable the recovery of selectable markers.   

## Supplementary Material

Supplemental File 1

## Supplementary Material

Supplemental File 2

## Supplementary Material

Supplemental File 3
