# Supplementary material for: A modular and optimized single marker system for generating Trypanosoma brucei cell lines expressing T7 RNA polymerase and the tetracycline repressor
Source: Open Biol. 2012 Feb;2(2):110037. doi: 10.1098/rsob.110037 (PMC3352093; doi:10.1098/rsob.110037)
Supplement: Supplemental File 3 [file rsob110037-s3.pdf]

Loading control for figure 2A

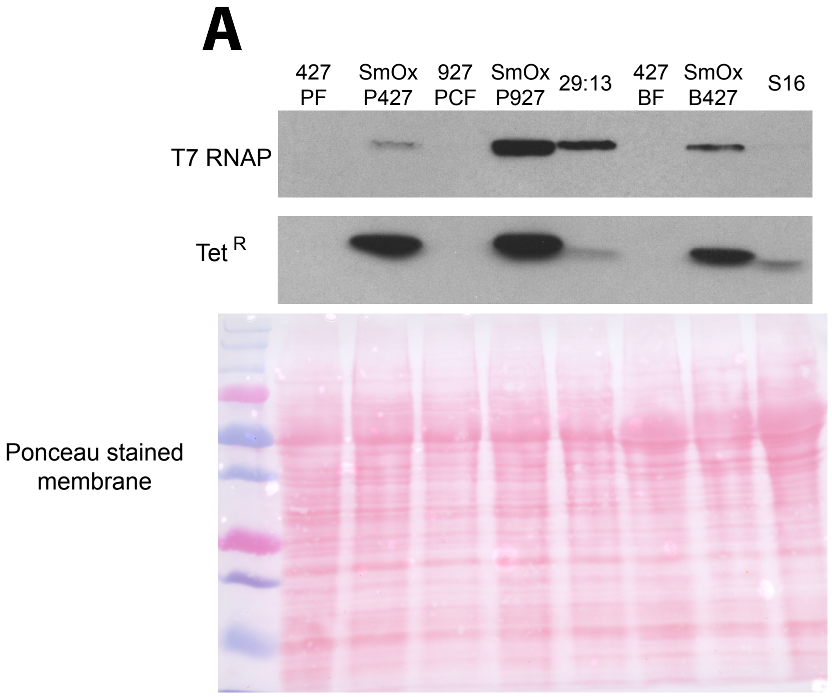

Loading control for Figure 4A

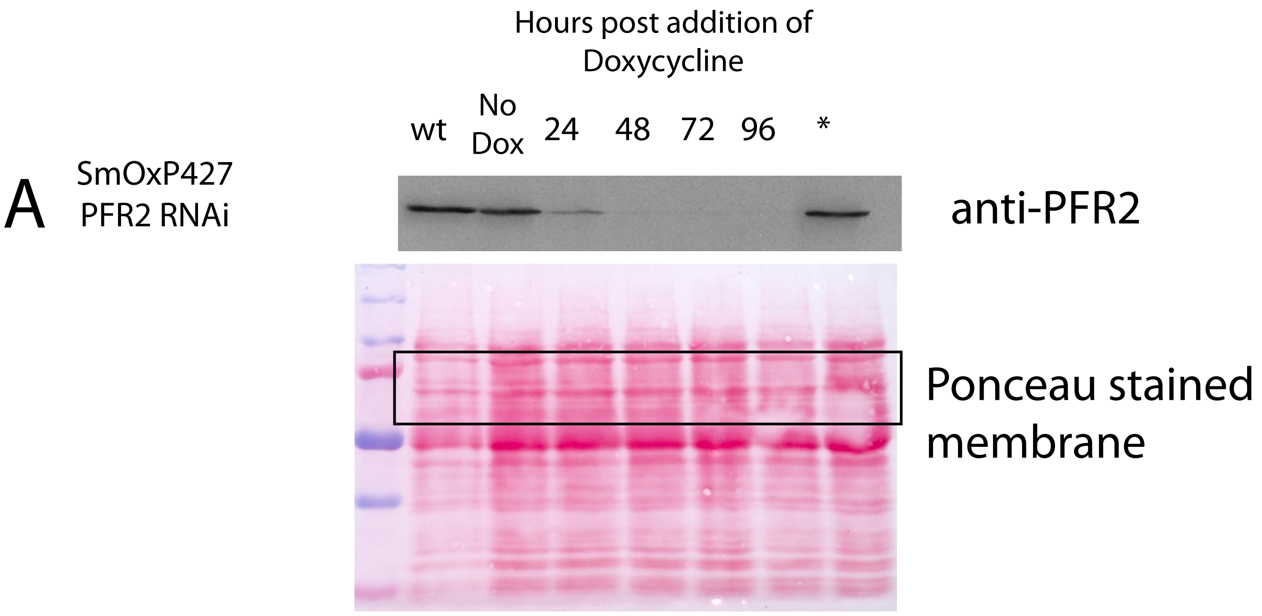

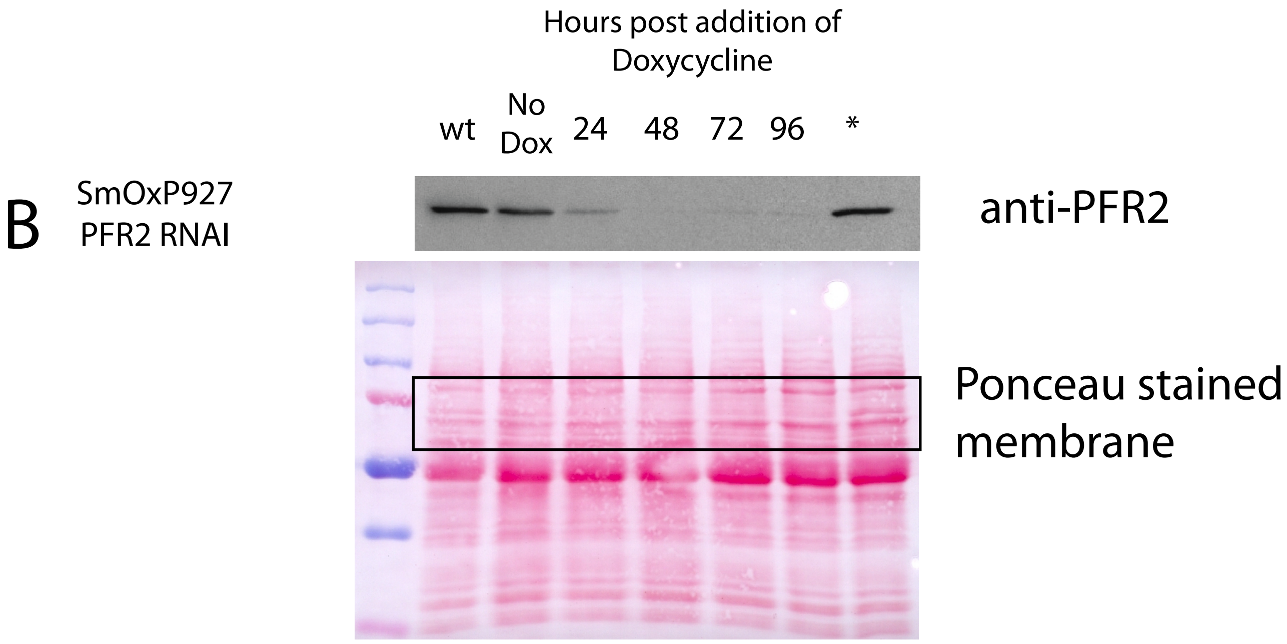

In both cases the black box indicates the area of the membrane shown in the western blot.
